# The Glasgow prognosis score is unsuitable for stroke prediction in infectious endocarditis

**DOI:** 10.1590/1806-9282.20240779

**Published:** 2024-09-16

**Authors:** Josef Finsterer

**Affiliations:** 1 Neurology and Neurophysiology Center – Vienna, Austria.

Dear Editor,

We read with interest the article by Aydin et al. about a study of 80 patients with bacterial endocarditis, diagnosed according to the Dukes criteria, in whom the Glasgow prognostic score (GPS) was retrospectively calculated^1^. The GPS did not differ between patients with (group 1) and without (group 2) stroke^1^. The GPS had a sensitivity of 82% and a specificity of 58% in predicting ischemic stroke^1^. In multivariate analysis, age, renal failure, and GPS predicted ischemic stroke^1^. The study is attractive but raises concerns that should be discussed.

The first point is that GPS uses only two serum parameters [albumin and C-reactive protein (CRP)] to predict outcomes, but the risk of stroke in these patients is multicausal. Additionally, CRP and reduced albumin can have numerous causes, which is why the GPS is nonspecific. GPS has been particularly used to predict the outcome of malignancies, the effects of chemotherapy, and the outcome of traumatic brain injury^2^. CRP is a frequently determined serum parameter, but its elevation is nonspecific. CRP may be elevated in acute and chronic infections, malignancies, rheumatological disease, vasculitis due to obesity and overweight, sedentary lifestyle, cigarette smoking, and diabetes. A decrease in CRP can be caused by taking non-steroidal, anti-inflammatory drugs (NSAIDs) or statins^3^. Therefore, it is important to know whether the history of the included patients was positive for any of these diseases and which medications the included patients were taking regularly. Like CRP, reduced albumin is also a non-specific finding. Since albumin is mainly produced in the liver, all types of liver disease that are associated with reduced albumin production lead to a decrease in serum albumin levels. Any disease that involves increased protein secretion, such as diarrhea, malabsorption, short bowel syndrome, or kidney disease, can also potentially cause hypoalbuminemia. Based on these considerations, GPS can be used to predict stroke in endocarditis only if all different causes of CRP elevation and albumin reduction have been ruled out.

A second point is that patients with endocarditis may suffer not only from heart problems but also from numerous complications. These include heart failure, cardioembolism, renal disease, ischemic embolic stroke, brain abscess, heart valve damage, pulmonary embolism, conjunctival hemorrhage, meningitis, and sepsis^4^. Some of these diseases can also increase CRP and reduce albumin, so it can be difficult to distinguish whether abnormal GPS is due to endocarditis or its complications.

The third point relates to the scores themselves. Although scores may make everyday medical practice easier, they also have some shortcomings and pitfalls. Scores should not prevent thinking, reading, reasoning, and engaging critically with data, information, trends, and guidelines.

The fourth point is that the stroke mechanism in patients with endocarditis may not only be cardioembolic but may also be atherosclerotic or due to coagulopathy, particularly when the endocarditis is associated with sepsis and therefore coagulopathy.

Since CRP and albumin can fluctuate during hospitalization, we should know whether the highest measured values or average values were used for the GPS calculation. We should also know how GPS changes during hospitalization. Diagnosis of cardioembolic stroke using cerebral CT (CCT) alone is inappropriate because CCT can easily miss small disseminated embolic strokes.

Because GPS results are nonspecific, they should not be used as a predictor of endocarditis outcomes. Patients with complaints suggestive of endocarditis should undergo a thorough examination of their medical history, a thorough clinical examination, and appropriate instrumental examinations. If all indications are considered, endocarditis should not be missed, and scores that are often created to stop thinking can be discarded.
